# Neoductgenesis in Ductal Carcinoma In Situ Coexists with Morphological Abnormalities Characteristic for More Aggressive Tumor Biology

**DOI:** 10.3390/diagnostics13040787

**Published:** 2023-02-19

**Authors:** Agnieszka Łazarczyk, Joanna Streb, Przemysław Hałubiec, Anna Streb-Smoleń, Robert Jach, Diana Hodorowicz-Zaniewska, Elżbieta Łuczyńska, Joanna Szpor

**Affiliations:** 1 Department of Pathomorphology, Jagiellonian University Medical College, 16 Grzegórzecka Street, 31-531 Cracow, Poland; 2 Department of Oncology, Jagiellonian University Medical College, 50 Kopernika Street, 31-501 Cracow, Poland; 3 University Hospital, 2 Jakubowskiego Street, 30-688 Cracow, Poland; 4 Department of Oncology, Maria Sklodowska-Curie National Research Institute of Oncology, 11 Gancarska Street, 31-115 Cracow, Poland; 5 Department of Gynecology and Obstetrics, Jagiellonian University Medical College, 23 Kopernika Street, 31-501 Cracow, Poland; 6 General, Oncological, and Gastrointestinal Surgery, Jagiellonian University Medical College, 2 Jakubowskiego Street, 30-688 Cracow, Poland; 7 Department of Electroradiology, Jagiellonian University Medical College, 12 Michałowskiego Street, 31-126 Cracow, Poland

**Keywords:** neoductgenesis, ductal carcinoma in situ, breast cancer, prognostic factors

## Abstract

Ductal carcinoma in situ (DCIS) is a non-invasive form of breast cancer that is generally indolent, however, could advance to invasive carcinoma in more than one-third of cases if left untreated. Thus, there is continuous research to find DCIS characteristics that would enable clinicians to decide if it could be left without intensive treatment. Neoductgenesis (i.e., formation of the new duct of improper morphology) is a promising, but still not sufficiently evaluated indicator of future tumor invasiveness. We gathered data from 96 cases of DCIS (histopathological, clinical, and radiological) to assess the relationship between the neoductgenesis and well-established features of high-risk tumor behavior. Furthermore, our intention was to determine which degree of neoductgenesis should be considered clinically significant. Our major finding was that neoductgenesis is strictly related to other characteristics that indicate the invasive potential of the tumor and, to achieve more accurate prediction, neoductgenesis should be accordingly recognized to less strict criteria. Therefore, we conclude that neoductgenesis is another important revelator of tumor malignancy and that it requires further investigation during prospective controlled trials.

## 1. Introduction

Ductal carcinoma in situ (DCIS) is a non-invasive form of breast cancer with an increasing number of diagnosed cases due to the use of mammography (MMG) screening (DCIS accounts for 20–25% of the lesions detected on screening MMG). The majority of these lesions are indolent; however, it is estimated that about 40% of them will convert to invasive carcinoma if left untreated. Some of these tumors might not require intensive treatment, which is very burdensome for patients. Therefore, it is important to look for prognostic factors that indicate high-risk changes requiring specific forms of treatment, such as surgery (including, if necessary, a choice between Breast Conserving Surgery (BCS) and mastectomy), radiotherapy, and hormonotherapy [1,2,3]. The most important issue is to establish the association between the tumor image in breast imaging and the histopathological features visible in the biopsy. DCIS includes a diverse spectrum of lesions of various morphologies, grades, and clinical presentations, but it constitutes 90% of breast cancer precursors. Independent predictors include the presence of comedo necrosis, micropapillary architecture, the younger age of the patient, and a strong family history of breast cancer [2].

Tabar et al. (2004), analyzing breast carcinomas with a diameter of 1–14 mm, showed a significantly worse prognosis in patients with casting-type calcifications in MMG. Microscopic examination of these tumors revealed the presence of a large number of oddly shaped, cancerous ducts in a tightly twisted arrangement. He called the phenomenon neoductgenesis [4].

Zhou et al. (2014) proposed histological criteria to define neoductgenesis as a marker of more aggressive forms of breast cancer. In this classification, the concentration of duct-like structures and loss of normal ductal-lobular architecture, lymphocytic infiltration, and fibrosis-like thickening of the periductal stroma are assessed. Each of these features is rated on a scale of 0–2, where 0 means no feature, 1 medium intensity, and 2 high intensity, considering the sum of points 5–6 as a determinant of neoductgenesis. Then, they showed that the appearance of neoductgenesis is correlated with the presence of malignant type microcalcifications and with the overexpression of HER2 [5]. However, after establishing a larger cohort of patients, it turned out that few DCIS met the criteria for neoductgenesis according to scores 5–6 (17 of 458), so researchers decided to change the criteria, considering neoductgenesis in the range of 4 to 6 points. However, this long-term cohort revealed that, while neoductgenesis and casting-type calcifications correlate with features of more aggressive tumor biology, they were associated with a non-significant lower risk of invasive IBE (ipsilateral breast events) [6].

The purpose of our study is to assess DCIS cases diagnosed in our center in terms of the presence of signs of neoductgenesis, to present the distribution of morphological features assessed on the scale proposed by Zhou et al. We divided DCIS cases according to the two proposed cut-off points for the presence of neoductgenesis. We evaluated their relationship with histological, radiological, and clinical features to determine which cut-off point gives us the most information about the aggressive behavior of the tumor.

## 2. Materials and Methods

**Figure 1:**
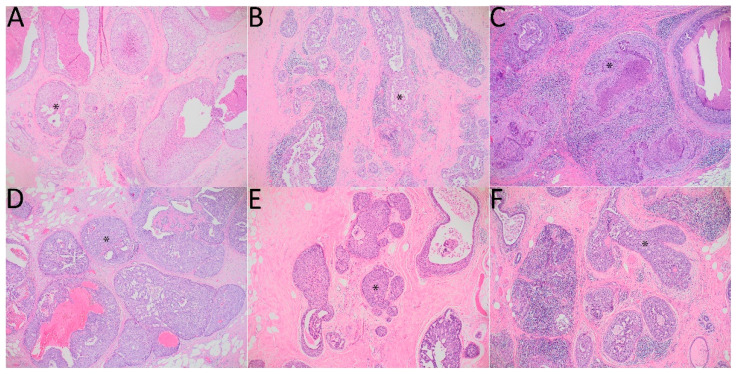
DCIS with signs of neoductgenesis. Hematoxylin-eosin stain. Magnification 100×. An asterisk (*) in each panel was added to indicate an example of the pathological duct. (**A**): focal concentration of duct-like structures and focal loss of normal ductal-lobular architecture–1 point, mild periductal lymphocytic infiltration–1 point, little fibrosis-like thickening of the periductal stroma–1 point, total score–3 points; (**B**): focal concentration of duct-like structures and focal loss of normal ductal-lobular architecture–1 point, intense periductal lymphocytic infiltration–2 points, no fibrosis-like thickening of the periductal stroma–0 points, total score: 3 points; (**C**): focal concentration of duct-like structures and focal loss of normal ductal-lobular architecture–1 point, no periductal lymphocytic infiltration–0 points, much fibrosis-like thickening of the periductal stroma–2 points, total score–3 points; (**D**): general concentration of duct-like structures and loss of normal ductal-lobular architecture–2 points, mild periductal lymphocytic infiltration–1 point, little fibrosis-like thickening of the periductal stroma–1 point, total points–4 points; (**E**), (**F**): focal concentration of duct-like structures and focal loss of normal ductal-lobular architecture–1 point, intense periductal lymphocytic infiltration–2 points, much fibrosis-like thickening of the periductal stroma–2 points, total score–5 points.

The study investigated 96 cases of primary DCIS diagnosed from August 2013 to May 2021 that were retrospectively evaluated. The gender distribution of the patients was 95 females and 1 male. The original histopathological report was the basis for inclusion in the study. The exclusion criteria for the study were the coexistence of invasive cancer and the prior diagnosis of invasive cancer in the same breast. The material consisted of routinely processed, formalin-fixed, and paraffin-embedded primary DCIS. Nuclear grade, architectural pattern, and other histological features of DCIS were evaluated according to College of American Pathologists protocols [7]. Immunohistochemistry for estrogen receptor (ER) and progesterone receptor (PR) was performed according to the protocol routinely used in our laboratory. The primary antibodies used in the study were manufactured by Novocastra (Leica Biosystems, Germany) for ER and Dako (USA) for PR. Positive expression of ER and PR was established when ≥1% of tumor cells showed positive immunostaining. The slides were evaluated by a histopathologist experienced in breast diagnosis (J.Sz.) and A.Ł. The features of neoductgenesis were evaluated based on the classification proposed by Zhou et al. (2014) [5]. The neoductgenesis cut-off point was established according to the two publications by Zhou et al. (2014, 2017), recognizing 5–6 [5] and 4–6 [6], respectively. Samples of microscopic images of neoductgenesis with scores assigned according to the system proposed by Zhou et al. are presented in Figure 1.

The grades of the BI-RADS (Breast Imaging-Reporting and Data System) scale in USG and MMG were assessed according to the ACR system (American College for Radiology). The biopsy was taken for BI-RADS grades 4–5 [8].

Statistical analysis was performed with Statistica 13.3 software (Statsoft Inc., Tulsa, OK, USA). Categorical data are presented as frequencies (N) and proportions (%), and interval data as mean ± SD. In the absence of any data, the case was not included in the analysis for a given variable. Comparisons of categorical variables were performed with the *χ*2 test (or with Fisher’s two-tailed test if any expected number was <5 or the size of the group N < 20). For interval variables, the Student’s t-test (or Welch’s test in the case of heterogeneity of variance in Levene’s test) was performed. If the Shapiro-Wilk test showed an abnormal distribution of the data, a nonparametric Mann-Whitney U test was used instead. Univariate logistic regression was used to determine the impact of neoductgenesis on prognostic factors in DCIS. A *p*-value below 0.05 was considered significant. The Benjamini–Hochberg (BH) procedure was used to correct for multiple testing (assuming FDR = 0.05) and an adjusted *p*^BH^ < 0.05 was considered significant.

The study was approved by the Jagiellonian University Bioethics Committee (1072.6120.289.2020 from 28 October 2020).

## 3. Results

### 3.1. Histopathological Features of Neoductgenesis

The distribution of the individual characteristics of neoductgenesis and the total score are shown in Table 1. The assessed DCIS cases presented varying prominence of the investigated characteristics in one tumor, especially in the case of lymphocytic infiltration. We chose the sites with the highest intensity of the trait in the observed area of the tumor. Among DCIS assessed by us, there was no case with the maximum score (i.e., 6 points).

Then, we divided the DCIS into those that met the criteria for neoductgenesis according to the two cut-off points. In the case of recognizing neoductgenesis as 4–6 points, twenty-seven (28.13%) cases were obtained, and nine (9.38%) cases obtained 5–6 points.

### 3.2. Scores 4–6 Considered as Neoductgenesis

DCIS showing signs of neoductgenesis (scores 4–6) significantly differed in terms of nuclear grade, having G3 significantly more frequently (*p*^BH^ = 0.011). Other histological features of malignancy were also more common in the neoductgenesis group, such as central necrosis (88.9% vs. 55.1%, *p*^BH^ = 0.021), lobular cancerization (88.9% vs. 48.8%, *p*^BH^ = 0.017) and ductal spread (96.3% vs. 67.7%, *p*^BH^ = 0.021). The cribriform histological type (33.3% in the neoductgenesis group to 65.2% without, *p*^BH^ = 0.024) and PR positive (32.0% vs. 68.5%, respectively; *p*^BH^ = 0.018) coexisted significantly less frequently. The neoductgenesis group was also characterized by larger tumor sizes (27.37 ± 18.31 mm vs. 12.30 ± 11.06 mm, *p*^BH^ < 0.001) and lower ER (33.7 ± 39.6% vs. 64.1 ± 37.1%, *p*^BH^ = 0.021) and PR (19.2 ± 33.5% vs. 38.2 ± 37.0%, *p*^BH^ = 0.028) expressions. Detailed data on histological characteristics are presented in Table 2.

In the context of radiological data, DCIS with neoductgenesis as scores 4–6 showed larger size on ultrasonography (USG; 23.4 ± 10.6 mm vs. 14.8 ± 9.6 mm, *p*^BH^ = 0.036) and MMG (40.9 ± 26.6 mm vs. 22.0 ± 21.3 mm, *p*^BH^ = 0.042). Detailed information on clinical data is presented in Table 3.

### 3.3. Scores 5–6 Considered as Neoductgenesis

In the case of neoductgenesis as scores 5–6, none of the examined parameters turned out to be significant after the B-H procedure. However, without the B-H procedure, a higher nuclear grade (percentage of G3) was present in the neoductgenesis group (77.8% vs. 34.5%, *p* = 0.039). Among the histological types, the comedo type (44.4% vs. 12.6%, *p* = 0.031) was more common in the group with neoductgenesis, and the cribriform type was less frequent (22.2% vs. 59.8%, *p* = 0.039). Lobular cancerization was much more frequent (88.9% vs. 78.2%, p = 0.033) and the mean diameter of the tumor was significantly higher (24.3 ± 18.3 mm vs. 15.7 ± 15.2 mm, *p* = 0.011). The percentage of PR positive (12.5% vs. 62.0%, *p* = 0.018) and ER positive (50.0% vs. 84.5%, *p* = 0.038) and the level of PR expression expressed as a percentage (1.3 ± 3.5% vs. 35.7 ± 37.2%, *p* = 0.009) were significantly lower. Detailed data on the distribution of histological features are shown in Table 4. In the case of clinical and radiological data, only the tumor diameter in USG showed a significant difference (30.8 ± 14.3 mm vs. 16.3 ± 9.0 mm, *p* = 0.033). The exact data are presented in Table 5.

### 3.4. The Univariate Logistic Regression Showing the Predictive Value of Neoductgenesis at Different Cut-Off Points

Univariate logistic regression showed that neoductgenesis as scores 4–6 may be a predictor of many histopathological characteristics associated with a worse prognosis. The presence of neoductgenesis as scores 4–6 was associated with a higher odds of central necrosis OR = 6.53 (95%CI = 1.80–23.72, *p*^BH^ = 0.008), lobular cancerization OR = 4.71 (95%CI = 1.80–23.72, *p*^BH^ = 0.012), ductal spread OR = 12.44 (95%CI = 1.58–97.66, *p*^BH^ = 0.027) and G3 OR = 5.26 (95%CI = 2.02–13.73, *p*^BH^ = 0.006). Moreover, the odds of a positive PR decreased, OR = 0.22 (95%CI = 0.08–0.60, *p*^BH^ = 0.008), indicating a worse prognosis. Considering the clinical data, the odds of using hormone therapy OR = 0.25 (95%CI = 0.08–0.74, *p*^BH^ = 0.036) decreased, which resulted from lower receptor expression in this group.

The univariate logistic regression with neoductgenesis as scores 5–6 did not result in any statistically significant outcome after the B-H procedure. Without this correction, it turned out to be a possible predictor of microinvasion, OR = 4.88 (95%CI = 1.02–23.32, *p* = 0.045), an important indicator of DCIS aggressiveness. Detailed data on the models can be found in Table 6.

## 4. Discussion

In our investigation, we evaluated the primary DCIS for the presence of histological signs of neoductgenesis. Then, we divided them into groups using two cut-off points. By the original approach, considering 5–6 points to indicate neoductgenesis, 9.4% of the cases met the criteria. On the contrary, considering 4–6 points as neoductgenesis (similarly to the classification proposed by Zhou et al. [5]), it was determined in 28.1% of DCIS cases. We evaluated the relationship between the features of neoductgenesis and established histological indicators of malignancy, attempting to determine which criterium properly reflects the tumor biology.

Our major finding was that neoductgenesis as scores 4–6 could be superior in determining the cases that show multiple other histological signs of malignancy. Nonetheless, tumors with neoductgenesis determined due to the score 5–6 exhibited microinvasion more frequently.

In the first study by Zhou et al. (2014), the incidence of neoductgenesis as 5–6 was 31.1% [5]. This is a result visibly different from ours; however, their group only consisted of cases with G2 and G3 grade tumors, without G1. As in our study, there was a statistically significant correlation between grade and the incidence of neoductgenesis (G3 coexistence with neoductgenesis as score 5–6) and expression of PR and ER (i.e., significantly lower in the neoductgenesis groups) [5]. In our study, we showed a significant difference in the grading distribution between cases with or without neoductgenesis, especially for G3. In the cohort recruited in the Zhou et al. second study, they only obtained 3.7% of cases of neoductgenesis, which was the reason for changing the neoductgenesis criterion to the scores 4–6. After the revision of criteria, the incidence of DCIS with neoductgenesis increased to 9.8%. Comparing other parameters, as in our study, the difference in grading (with a predominance of G3 in the neoductgenesis group) and a lower PR expression emerged. In the case of ER-positivity, Zhou et al. (2017) obtained a significant relationship, in contrast our results. However, we adopted a different definition of positive receptor expression (≥10% in Zhou et al. study vs. ≥1% in our analysis). We found the difference in receptor expression, which was significantly lower in the group with neoductgenesis as scores 4–6 [6].

Zombori et al. (2017) assessed DCIS that showed the presence of comedo necrosis to investigate the presence of an elastic layer that should surround the normal ducts. It turned out that it did not occur around unclassifiable structures (i.e., structures that were not considered to be ducts or lobules). The authors of the study hypothesized that these structures resulted from neoductgenesis. This is an argument in favor of the hypothesis that neoductgenesis is the formation of new pathological ducts that grow into the stroma. Defining neoductgenesis as scores 5–6, they showed that 25.5% of the cases assessed by them met the criteria. The difference in the frequency of the phenomenon, compared to our results, was due to the fact that Zombri et al. evaluated DCIS that all had comedo necrosis, which, according to our analysis, is significantly more common in cases showing signs of neoductgenesis and other unfavourable prognostic characteristics, such as G3, and some cases coexisting with the invasive component [9].

Tot presented the sick lobe theory, which assumed that whole lobe abnormalities are involved in epithelial cell transformation. The formation of malignant lesions is usually associated with aberrant lobularization and/or aberrant branching in a sick lobe. According to the author, low-grade DCIS shows features of the lobularization process, while high-grade DCIS shows features of arborization. The mesenchyme surrounding the new ducts shows overexpression of tenascin C, which appears to be a sensitive indicator of arborization within the lobe. These assumptions gave rise to the theory of neoductgenesis, which described the formation of DCIS as associated with a worse prognosis [10]. Tabár et al. performed a mammographic evaluation of invasive breast cancers of 1–14 mm in diameter and then assessed histological grade, lymph node status, and 24-year survival in a group of 714 women. According to their elaboration, tumors containing casting-type calcifications were associated with an increased chance of poorer histological grade, positive lymph nodes status, and a higher risk of death from breast cancer. The authors suggested that the process of neoductgenesis is a possible explanation for the formation of casting-type calcifications [4]. On examination of the section, subgross (3D) histology samples obtained from their patients showed a tortuous, unnaturally densely packed cluster of duct-like structures that were filled with cancer cells. They were accompanied by an intense inflammatory infiltrate [11]. This suggests that DCIS that exhibit signs of neoductgenesis, have different biology, are associated with a worse prognosis, and their diagnosis will allow the segregation of cases requiring more intensive forms of treatment.

The purpose of our publication was to investigate how the neoductgenesis is associated with other histological and clinical characteristics of DCIS. Another purpose was to re-evaluate the cut-off points proposed by Zhou et al. [5,6]. An important update stemming from our elaboration is establishing the decisive cut-off of 4–6 points as a clinically pertinent criterion for neoductgenesis (being of practical importance also due to the too low frequency of samples that received a sum of 5–6 points). To this, we demonstrated that neoductgenesis classified in such a way coexists with microarchitectural abnormalities typical for tumors with a high invasive potential. Univariate logistic regression showed that neoductgenesis as scores 4–6 may predict the presence of many factors of poor prognosis. There is a more than 5-fold significant increase (OR = 5.26) in the odds of G3 presence compared to G1–G2. This is a very important factor that indicates a worse prognosis and an increase in tumor aggressiveness [12,13]. A notable negative prognostic factor is also the presence of comedo necrosis and low expression of hormone receptors, which increase the risk of disease recurrence [14]. The odds of the presence of ductal spread and lobular cancerization significantly increase, which is associated with signs of neoductgenesis, pathological ducts filled with cancer that eventually penetrate the lobules and fill them with tumor cells [9]. Importantly, tumors diagnosed with neoductgenes were significantly larger at the time of surgery. In the case of neoductgenesis as score 5–6, no result was significant after the B-H procedure. However, interestingly, without this adjustment, statistical significance was shown for an increase in the odds of microinvasion. This is a particularly important parameter, as it shows that DCIS has already begun the process of entering the invasive form and manifests a worse prognosis [15].

A particularly important outcome was that there was a significant difference in the size of the neoplasms in imaging tests. DCISs with signs of neoductgenesis as 4–6 were larger on imaging examinations, resulting in their easier detection [16]. Radiological and pathomorphological correlation (assessment of tumor biopsy) is extremely important in establishing the malignancy of lesions [17].

## 5. Conclusions

We conclude that neoductgenesis is an important revelator of tumor malignancy. It seems likely that defining neoductgenesis as scores 4–6 from Zhou classification would result in a more accurate determination of tumors with markers of malignant potential and lower expression of PR. Application of the definition that adopted scores 5–6 as neoductgenesis (which was indeed better associated with microinvasion) could result in the omission of some cases of tumors with malignant potential, and thus should be defied. However, to unambiguously determine if neoductgenesis should be considered during clinical decisions, prospective controlled trials should be performed.

The limitations of this study are the relatively small group of patients who underwent pathomorphological diagnostics in our center and met the inclusion criteria for the study. The performed analyses were retrospective, and information on clinical data was obtained from patients’ medical records. Moreover, the traditional statistical analyses used in this work are frequently considered as having worse accuracy than the novel approaches utilizing the methods of machine-learning, and they should be inarguably preferred in the upcoming research. For this reason, we cannot unambiguously conclude about the cause-effect relationships between the studied variables.

## Figures and Tables

**Table 1:** Histopathological signs of neoductgenesis, the number and percentage distribution of individual features and the distribution of the sum of points obtained in the classification.

Characteristic		N = 96	%
Concentration of duct-like structures	No (0)	15	15.6
	Focal (1)	69	71.9
	General (2)	12	12.5
Lymphocytic infiltration	No (0)	32	33.3
	Mild (1)	48	50.0
	Intense (2)	16	16.7
Fibrosis-like thickening of the periductal stroma	No (0)	47	49.0
	Little (1)	27	28.1
	Much (2)	22	22.9
Total score	0	6	6.3
	1	20	20.8
	2	22	22.9
	3	21	21.9
	4	18	18.8
	5	9	9.4
	6	0	0

**Table 2:** Comparison of histological features in DCIS with or without neoductgenesis as scores 4–6.

Characteristic		Neoductgenesis		*p*-Value/*p*^BH^
		Absent, N = 69	Present, N = 27	
Nuclear grade (N, %)	G1	4 (5.8)	0 (0)	0.001/0.011
	G2	46 (66.7)	9 (33.3)	
	G3	19 (27.5)	18 (66.7)	
Histological type (N, %)	Comedo	7 (10.1)	8 (29.6)	0.028/–
	Solid	50 (72.5)	21 (77.8)	0.783/–
	Cribiform	45 (65.2)	9 (33.3)	0.009/0.024
	Micropapillary	23 (33.3)	9 (33.3)	0.810/–
	Papillary	15 (21.7)	3 (11.1)	0.363/–
	Apocrine	4 (5.8)	3 (11.1)	0.397/–
	Clinging	1 (1.5)	0 (0)	1.000/–
	Spindle cell	1 (1.5)	0 (0)	1.000/–
Central necrosis (N, %)		38 (55.1)	24 (88.9)	0.004/0.021
Lymph nodes micrometastases (N, %)		1 (2.1)	0 (0%)	1.000/–
Ductal spread (N, %)		46 (67.7)	26 (96.3)	0.007/0.021
Lobular cancerization (N, %)		29 (42.7)	21 (77.8)	0.004/0.017
Microcalcifications (N, %)		52 (75.4)	24 (88.9)	0.235
Paget disease (N, %)		9 (13.0)	2 (7.4)	0.723
Microinvasion (N, %)		5 (7.4)	6 (22.2)	0.070
Tumor size (mm)		12.3 ± 11.1	27.4 ± 18.3	<0.001/<0.001
ER (%)		64.1 ± 37.1	33.7 ± 39.6	0.003/0.021
ER positive, (N, %)		47 (87.0)	17 (68.0)	0.064
PR (%)		38.2 ± 37.0	19.2 ± 33.5	0.012/0.028
PR positive, (N, %)		37 (68.5)	8 (32.0)	0.005/0.018

Abbreviations: ER—estrogen receptor; PR—progesterone receptor.

**Table 3:** Clinical and radiological characteristic of DCIS with or without neoductgenesis as scores 4–6.

Characteristic		Neoductgenesis		*p*-Value/*p*^BH^
		Absent, N = 69	Present, N = 27	
**Clinical Data**				
Age at diagnosis (years)		55.5 ± 10.6	56.5 ± 12.0	0.557/–
The largest tumor size in physical examination (mm)		21.0 ± 11.8	28.3 ± 11.7	0.156/–
The palpability of the lesion	Yes (N, %)	10 (15.7)	9 (40.9)	0.106/–
	No (N, %)	33 (64.7)	12 (54.6)	
	Paget disease (N, %)	8 (15.7)	1 (4.56)	
Type of surgery	Mastectomy (N, %)	21 (39.6)	11 (47.8)	0.700/–
	BCT (N, %)	32 (60.4)	12 (52.2)	
Radiotherapy (N, %)		24 (53.3)	11 (47.8)	0.862/–
Hormonotherapy (N, %)		30 (65.2)	7 (31.8)	0.020/–
Family history of breast cancer (N, %)		11 (22.5)	3 (13.0)	0.525/–
**Radiological Data**				
Solid lesion in MMG (N, %)		9 (25.7)	10 (45.5)	0.211/–
BI-RADS USG (N, %)	1	10 (25.0)	6 (30.0)	0.285/–
	2	10 (25.0)	1 (5.0)	
	3	3 (7.5)	2 (10.0)	
	4	17 (42.5)	7 (35.0)	
	5	0 (0)	4 (20.0)	
BI-RADS MMG (N, %)	0	2 (5.1)	2 (9.1)	0.028/0.056
	1	5 (12.8)	0 (0)	
	2	3 (7.7)	0 (0)	
	3	3 (7.7)	1 (4.6)	
	4	25 (64.1)	13 (59.1)	
	5	1 (2.6)	6 (27.3)	
The largest tumor size in USG (mm)		14.8 ± 9.6	23.4 ± 10.6	0.006/0.036
The largest tumor size in MMG (mm)		22.0 ± 21.3	40.9 ± 26.6	0.014/0.042
Microcalcifications in MMG (N, %)		28 (75.7)	17 (77.3)	0.860/–

Abbreviations: BI-RADS—Breast Imaging-Reporting and Data System; BCT—breast-conserving therapy; MMG—mammography; USG—ultrasonography.

**Table 4:** Comparison of histological features in DCIS with or without neoductgenesis as scores 5–6.

Characteristic		Neoductgenesis		*p*-Value/*p*^BH^
		Absent, N = 87	Present, N = 9	
Nuclear grade (N, %)	G1	4 (4.6)	0 (0)	0.039/–
	G2	53 (60.9)	2 (22.2)	
	G3	30 (34.5)	7 (77.8)	
Histological type (N, %)	Comedo	11 (12.6)	4 (44.4)	0.031/–
	Solid	63 (72.4)	8 (88.9)	0.438/–
	Cribiform	52 (59.8)	2 (22.2)	0.039/–
	Micropapillary	30 (34.5)	2 (22.2)	0.713/–
	Papillary	18 (20.7)	0 (0)	0.201/–
	Apocrine	6 (6.9)	1 (11.1)	0.510/–
	Clinging	1 (1.2)	0 (0)	1.000/–
	Spindle cell	1 (1.2)	0 (0)	1.000/–
Comedo necrosis (N, %)		38 (55.1)	54 (62.1)	0.152/–
Lymph nodes micrometastases (N, %)		1 (2.1)	1 (1.6)	1.000/–
Ductal spread (N, %)		46 (67.7)	63 (73.3)	0.108/–
Lobular cancerization (N, %)		29 (42.7)	42 (48.8)	0.033/–
Microcalcifications (N, %)		52 (75.4)	68 (78.2)	0.680/–
Paget disease (N, %)		9 (13.0)	11 (12.6)	0.592/–
Microinvasion (N, %)		5 (7.4)	8 (9.3)	0.067/–
Tumor size (mm)		12.3 ± 11.06	15.7 ± 15.2	0.011/–
ER (%)		64.1 ± 37.1	57.1 ± 39.4	0.078/–
ER positive (N, %)		47 (87.0)	60 (84.5)	0.038/–
PR (%)		38.2 ± 37.0	35.7 ± 37.2	0.009/–
PR positive (N, %)		37 (68.5)	44 (62.0)	0.018/–

**Table 5:** Clinical and radiological characteristic of DCIS with or without neoductgenesis as scores 5–6.

Characteristic		Neoductgenesis		*p*-Value/*p*^BH^
		Absent, N = 69	Present, N = 27	
**Clinical Data**				
Age at diagnosis (years)		56.0 ± 10.9	50.1 ± 10.7	0.126/–
The largest tumor size in physical examination (mm)		23.0 ± 11.7	33.3 ± 11.6	0.211/–
The palpability of the lesion	Yes (N, %)	42 (62.7)	3 (50.0)	0.302/–
	No (N, %)	16 (23.9)	3 (50.0)	
	Paget disease (N, %)	9 (13.4)	0 (0)	
Type of surgery	Mastectomy (N, %)	29 (42.7)	5 (62.5)	1.000/–
	BCT (N, %)	39 (57.4)	3 (37.5)	
Radiotherapy (N, %)		31 (50.8)	4 (57.1)	1.000/–
Hormonotherapy (N, %)		35 (57.4)	2 (28.6)	0.233/–
Family history of breast cancer (N, %)		13 (20.0)	1 (14.3)	1.000/–
**Radiological Data**				
Solid lesion in MMG (N, %)		17 (33.3)	2 (33.3)	1.000/–
BI-RADS USG	1	15 (27.8)	1 (16.7)	0.654/–
	2	10 (18.5)	1 (16.7)	
	3	5 (9.3)	0 (0.0)	
	4	20 (37.0)	4 (66.7)	
	5	4 (6.7)	0 (0.0)	
BI-RADS MMG	0	3 (5.5)	0 (0.0)	0.383/–
	1	5 (9.1)	0 (0.0)	
	2	5 (12.8)	0 (0)	
	3	3 (5.5)	1 (4.6)	
	4	35 (63.6)	3 (50.0)	
	5	5 (9.1)	2 (33.3)	
The largest tumor size in USG (mm)		16.3 ± 9.0	30.8 ± 14.3	0.033/–
The largest tumor size in MMG (mm)		29.1 ± 25.8	31.7 ± 14.6	0.476/–
Microcalcifications in MMG (N, %)		40 (75.5)	5 (83.3)	1.000/–

**Table 6:** Univariate logistic regression model that predicts the OR of distinct histological, radiological and clinical characteristics of DCIS depending on the presence of neoductgenesis as scores 4–6 and neoductgenesis as scores 5–6.

Characteristic	Neoductgenesis as Scores 4–6			Neoductgenesis as Scores 5–6		
	OR	95%CI	*p*-Value/*p*^BH^	OR	95%CI	*p*-Value/*p*^BH^
**Pathology**						
Comedo necrosis	6.53	1.80–23.72	0.004/0.008	4.89	0.59–40.87	0.143/–
Ductal spread	12.44	1.58–97.66	0.017/0.027	–	–	0.997/–
Lobular cancerization	4.71	1.69–13.14	0.003/0.012	8.38	1.01–69.93	0.049/–
Microcalcifications	2.62	0.70–9.78	0.153/–	2.24	0.26–19.00	0.461/–
Microinvasion	3.60	0.995–13.02	0.051/–	4.88	1.02–23.32	0.045/–
ER-positive	0.32	0.10–1.01	0.051/–	0.18	0.04–0.85	0.03/–
PR-positive	0.22	0.08–0.60	0.003/0.008	0.08	0.01–0.75	0.026/–
Grading G3	5.26	2.02–13.73	<0.001/0.006	6.65	1.3–34.03	0.023/–
**Radiology**						
BI-RADS 4–5 (Biopsies indicators)	5.09	0.59–43.69	0.138/–	–	–	0.998/–
Solid lesion in MMG	2.41	0.78–7.50	0.128/–	–	–	1.000/–
**Clinical**						
Symptomatic tumor (palpable lesions and Paget disease)	1.53	0.55–4.22	0.414/–	1.68	0.32–8.97	0.544/–
Solid lesion in MMG	2.41	0.78–7.50	0.128/–	–	–	1.000/–
Hormonotherapy	0.25	0.08–0.74	0.012/0.036	0.3	0.05–1.65	0.166/–

Abbreviations: CI—confidence interval; OR—odds ratio.

## Data Availability

The data presented in this study are available on request from the corresponding author. The data are not publicly available due to privacy restrictions.

## References

[1] Badve S.S., Gökmen-Polar Y. (2019). Ductal carcinoma in situ of breast: Update 2019. Pathology.

[2] Groen E.J., Elshof L.E., Visser L.L., Rutgers E.J.T., Winter-Warnars H.A.O., Lips E.H., Wesseling J. (2017). Finding the balance between over- and under-treatment of ductal carcinoma in situ (DCIS). Breast.

[3] Martínez-Pérez C., Turnbull A.K., Ekatah G.E., Arthur L.M., Sims A.H., Thomas J.S., Dixon J.M. (2017). Current treatment trends and the need for better predictive tools in the management of ductal carcinoma in situ of the breast. Cancer Treat. Rev..

[4] Tabar L., Tony Chen H.H., Amy Yen M.F., Tot T., Tung T.H., Chen L.S., Chiu Y.H., Duffy S.W., Smith R.A. (2004). Mammographic tumor features can predict long-term outcomes reliably in women with 1–14-mm invasive breast carcinoma. Cancer.

[5] Zhou W., Sollie T., Tot T., Pinder S.E., Amini R.M., Blomqvist C., Fjällskog M.L., Christensson G., Abdsaleh S., Wärnberg F. (2014). Breast Cancer with Neoductgenesis: Histopathological Criteria and Its Correlation with Mammographic and Tumour Features. Int. J. Breast Cancer.

[6] Zhou W., Sollie T., Tot T., Blomqvist C., Abdsaleh S., Liljegren G., Wärnberg F. (2017). Ductal Breast Carcinoma In Situ: Mammographic Features and Its Relation to Prognosis and Tumour Biology in a Population Based Cohort. Int. J. Breast Cancer.

[7] Lester S.C., Bose S., Chen Y.Y., Connolly J.L., de Baca M.E., Fitzgibbons P.L., Hayes D.F., Kleer C., O’Malley F.P., Page D.L. (2009). Protocol for the Examination of Specimens from Patients with Invasive Carcinoma of the Breast. Arch. Pathol. Lab. Med..

[8] Jakubowski W., Dobruch-Sobczak K., Migda B. (2012). Standards of the Polish Ultrasound Society—Update. Sonomammography examination. J. Ultrason..

[9] Zombori T., Cserni G. (2017). Elastic stains in the evaluation of DCIS with comedo necrosis in breast cancers. Virchows Arch..

[10] Tot T. (2005). DCIS, cytokeratins, and the theory of the sick lobe. Virchows Arch..

[11] Tabár L., Dean P.B., Yen A.M.F., Tarján M., Chiu S.Y.H., Chen S.L.S., Fann J.C.Y., Chen T.H.H. (2014). A Proposal to Unify the Classification of Breast and Prostate Cancers Based on the Anatomic Site of Cancer Origin and on Long-term Patient Outcome. Breast Cancer.

[12] Poller D.N., Barth A., Slamon D.J., Silverstein M.J., Gierson E.D., Coburn W.J., Waisman J.R., Gamagami P., Lewinsky B.S. (1995). Prognostic classification of breast ductal carcinoma-in-situ. Lancet.

[13] Ponti A., Ronco G., Lynge E., Tomatis M., Anttila A., Ascunce N., Broeders M., Bulliard J.L., Castellano I., Fitzpatrick P. (2019). Low-grade screen-detected ductal carcinoma in situ progresses more slowly than high-grade lesions: Evidence from an international multi-centre study. Breast Cancer Res. Treat..

[14] Roguljic A., Spagnoli G., Juretic A., Sarcevic B., Banovic M., Beketic Oreskovic L. (2018). Possible predictive role of cancer/testis antigens in breast ductal carcinoma in situ. Oncol. Lett..

[15] Champion C.D., Ren Y., Thomas S.M., Fayanju O.M., Rosenberger L.H., Greenup R.A., Menendez C.S., Hwang E.S., Plichta J.K. (2019). DCIS with Microinvasion: Is It In Situ or Invasive Disease?. Ann. Surg. Oncol..

[16] Harbeck N., Penault-Llorca F., Cortes J., Gnant M., Houssami N., Poortmans P., Ruddy K., Tsang J., Cardoso F. (2019). Breast cancer. Nat. Rev. Dis. Prim..

[17] Tot T., Tabár L. (2010). The role of radiological–pathological correlation in diagnosing early breast cancer: The pathologist’s perspective. Virchows Arch..

